# Humoral immune response to heat shock protein 60 of *Aggregatibacter actinomycetemcomitans* and cross-reactivity with malondialdehyde acetaldehyde-modified LDL

**DOI:** 10.1371/journal.pone.0230682

**Published:** 2020-03-25

**Authors:** Mikael Kyrklund, Mika Bildo, Ramin Akhi, Antti E. Nissinen, Pirkko Pussinen, Sohvi Hörkkö, Chunguang Wang

**Affiliations:** 1 Research Unit of Biomedicine, Medical Microbiology and Immunology, Faculty of Medicine, University of Oulu, Oulu, Finland; 2 Medical Research Center and Nordlab Oulu, University Hospital and University of Oulu, Oulu, Finland; 3 Oral and Maxillofacial Diseases, University of Helsinki and Helsinki University Hospital, Helsinki, Finland; 4 Minerva Foundation Institute for Medical Research, Helsinki, Finland; Instituto Butantan, BRAZIL

## Abstract

Atherosclerosis is a chronic inflammatory disease and major cause of mortality worldwide. One of the crucial steps for atherosclerotic plaque development is oxidation of low-density lipoprotein (LDL). Through the oxidation, highly immunogenic epitopes are created and the immune system is activated. Association between atherosclerosis and periodontal diseases is well documented, and one of the main oral pathogens common in periodontitis is *Aggregatibacter actinomycetemcomitans* (*Aa*). Heat shock protein 60 (HSP60) is an important virulence factor for *Aa* bacteria and a strong activator of the immune system. Cross-reactivity of HSP60 and oxidized LDL (OxLDL) antibodies could be a potential mechanism in the progression of atherosclerosis and one possible link between atherosclerosis and periodontitis. Human plasma samples from neonates and mothers were analyzed to determine if antibody titer to *Aa*-HSP60 protein is already present in newborns. Further objectives were to characterize antibody response in *Aa*-HSP60 immunized mice and to determine possible antibody cross-reaction with oxidized LDL. We demonstrated that newborns already have IgM antibody levels to *Aa*-HSP60. We also showed that in mice, *Aa*-HSP60 immunization provoked IgG and IgM antibody response not only to *Aa*-HSP60 but also to malondialdehyde acetaldehyde-modified LDL (MAA-LDL). Competition assay revealed that the antibodies were specific to *Aa*-HSP60 and cross-reacted with MAA-LDL. Our results suggest a possibility of molecular mimicry between *Aa*-HSP60 and MAA-LDL, making it intriguing to speculate on the role of HSP60 protein in atherosclerosis that manifests at young age.

## Introduction

Atherosclerosis is a chronic inflammatory disease in which both innate and adaptive immune systems play an important role. The disease starts early in life, becomes clinically manifest at older age, and associates strongly with cardiovascular disease (CVD), a major cause of death worldwide [1]. Atherosclerosis narrows the arteries due to the formation of plaque that accumulates in the arterial intima. Inside the intima, low-density lipoprotein (LDL) particles go through oxidative modification which creates oxidized LDL particles (OxLDL). Through oxidation, malondialdehyde-modified LDL (MDA-LDL) and further, malondialdehyde-acetaldehyde-modified LDL (MAA-LDL) particles are created [2]. The MAA adducts are highly immunogenic and known to be a target for human natural antibodies already in newborns [3].

The exact role of antibodies to OxLDL in atherosclerosis remains elusive. IgM antibodies to OxLDL are considered to have protective properties, whereas IgG antibodies to OxLDL are more heterogeneous but mainly considered pro-atherogenic [3–7]. IgM antibodies to OxLDL inhibit the cholesterol uptake of macrophages through scavenger receptors, explaining the atheroprotective properties of IgM antibodies [8]. It is hypothesized that low levels of MAA adduction are rapidly eliminated through the scavenger receptors with IgM stimulation. In chronic conditions and constant tissue injuries the levels of MAA-adducts are elevated, which could lead to a shift in the clearance of MAA adducts through the scavenger receptors by possibly activating B-cells and switching Ig class to IgG, which would further enhance the local inflammation [9].

*Aggregatibacter actinomycetemcomitans* (*Aa*) is one of the most studied oral pathogens associated with periodontitis, a chronic inflammatory disease affecting tooth supporting tissue and alveolar bone. Periodontitis is highly common in adult population and is known to associate with atherosclerosis [10]. Treatment of periodontitis significantly diminish the total load of oral pathogens that is associated with reduced serum inflammatory markers, improvements in endothelial dysfunction and reduction of the carotid intima-media thickness, which relate to the progression of atherosclerosis [11]. Although the exact mechanism linking these two chronic infections is yet to be known, many mechanisms have been proposed. Molecular mimicry and cross-reactivity of the antibodies is one of the hypotheses linking these two diseases.

Heat shock protein 60 (HSP60) is one of the main virulence factors of *A*. *actinomycetemcomitans*, a protein that has also been demonstrated to associate with atherosclerosis [12]. Under normal conditions, heat shock proteins have a variety of different functions: intracellular folding, transportation, and working as chaperons [13]. These proteins are vastly upregulated when cells are exposed to a stress factor, which leads further to T-cell activation. Heat shock proteins activate the immune system but their exact role in atherosclerosis remains unknown. In clinical studies, increased antibody titers to HSP60 have been shown to be linked with the severity of atherosclerosis [14]. Antibodies to HSP60 could work as autoantibodies accelerating the atherogenesis. Pre-existing immunity to HSP60 from various pathogens could cross-react with host natural HSP60 proteins causing the autoimmune reactions. HSPs are a group of proteins with high sequence similarity among species, from humans to bacteria, which explains the cross-reaction possibilities between host and pathogenic HSP60 proteins [15].

Antibodies to MAA-LDL and HSP60 are both associated with atherosclerosis and they are both produced under similar stressed conditions [13,16]. In our previous study, we identified natural IgM antibodies that recognize MAA-LDL from human umbilical cord blood [3]. We also cloned natural mouse monoclonal IgM antibody to MAA-LDL that bound to HSP60 of *Aa* bacteria [17]. In this study, we investigated whether human neonates have natural antibodies to Aa-HSP60 and whether the antibodies to Aa-HSP60 cross-react with MAA-LDL. Mouse experiments were also carried out to verify the possible cross-reactions between HSP 60 and MAA-LDL antibodies as cross-reaction could be one possible mechanism in the progression of atherosclerosis and a potential link between atherosclerosis and periodontitis.

## Materials and methods

### Human samples

Blood samples from neonates were collected from the umbilical cord immediately after delivery. In total, 13 pre-term blood samples (< 32 weeks of gestation) and 36 full-term blood samples (> 36 weeks of gestation) were used in this study. Venous blood samples from mothers were collected 24–48 h after delivery (48 samples). All the plasma samples were handled as previously reported [3], and IgM and IgG antibodies’ binding characteristics to *Aa*-HSP60, MAA-LDL and fish gelatin/phosphate-buffered saline (Fg-PBS) were tested and compared. Specific binding of neonates’ plasma IgM antibodies to *Aa*-HSP60 and MAA-LDL was tested with two points competitive chemiluminescence immunoassay. *Aa*-HSP60 and MAA-LDL (0 and 100 μg/mL) were incubated with plasma samples overnight at +4°C and immunoassay was performed as described below. The study was approved by the ethical committee of the Oulu University Hospital, Finland (195/2006) and informed written consent was obtained from each participant.

### Mice immunization

The study was approved by animal research ethics committee, Animal Experiment Board in Finland (ELLA), with the permit number ESAVI/9168/04.10.07/2014. Female C57BL/6J mice (n = 4, about 8 weeks-old) were used. The immunization was carried out as shown in supplemental Fig 1(S1 Fig) with the support of The Oulu Laboratory Animal Centre Research Infrastructure, University of Oulu, Finland. Primary immunization was injected subcutaneously with 50 μg of *Aa*-HSP60 protein diluted in 200 μL of saline. Intraperitoneal booster injections were done with 25 μg of *Aa*-HSP60 protein. Boosters were first given three times every two weeks, followed by three boosters given with 4–6 weeks intervals. No adjuvants were used in this study. The mice were on regular chow diet (4.4% fat and 0.02% cholesterol) throughout the study and were sacrificed one week after the last booster. At the endpoint of the study, mice were euthanized by carbon dioxide (CO2) and cervical dislocation. Blood sampling was performed with anesthesia by combination of Hypnorm^®^ (fentanyl citrate 0.079 mg/ml, fluanisone 2.5 mg/ml) and Dormicum^®^ (Midazolam 1.25 mg/ml) at 0.05–0.1ml/10g. Control blood samples were taken before the primary immunization, followed by regular blood collections throughout the study. Blood samples were collected from saphenous vein and the final blood samples were taken from vena cava after sacrifice. Pre-immunization blood samples are referred to as “week 0”, while “week 15” refers to the blood samples collected after the fourth booster injection.

**Fig 1:**
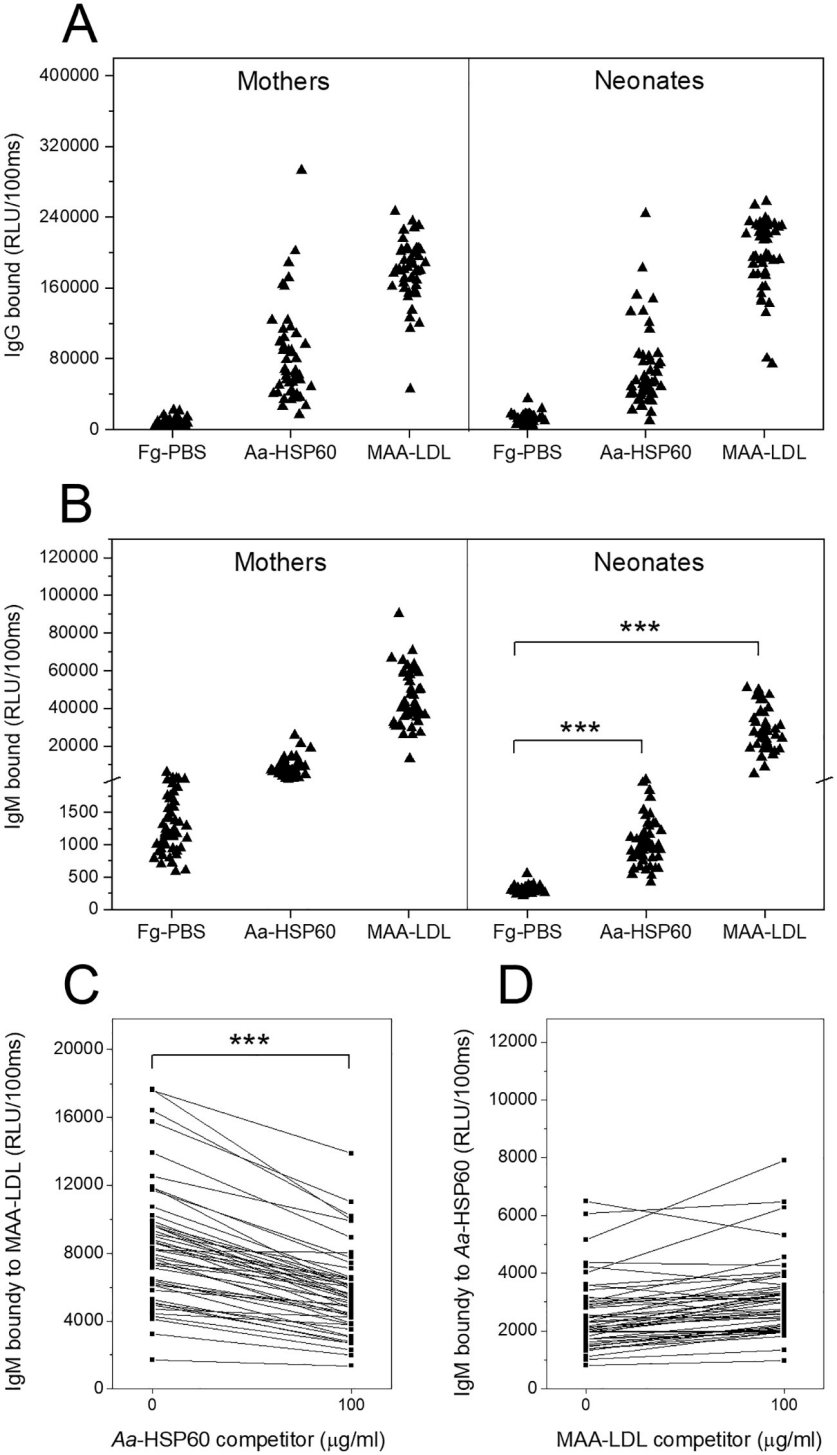
Human plasma IgG and IgM antibodies binding to *Aa*-HSP60 and MAA-LDL. Plasma IgG (A) and IgM (B) antibody levels to Fg-PBS (fish gelatin phosphate-buffered saline), *Aa*-HSP60, and MAA-LDL from mothers (n = 48) and neonates (n = 49) were measured. Antibody levels are shown as box-plots, representing 25%, 50% and 75% of the distribution, and whiskers representing 10% and 90% distribution of the values. The cross represents the maximum and minimum values and the solid diamonds are the mean values. The competitive immunoassay of neonates’ plasma IgM binding to MAA-LDL after competed with *Aa*-HSP60 (C) and IgM binding to *Aa*-HSP60 after competed with MAA-LDL (D) were shown. Comparative binding specificity was demonstrated in the absence (0 μg/mL) or presence (100 μg/mL) of the soluble competitors. The antibody binding is expressed in relative light units measured in 100 milliseconds (RLU/100ms). P-values less than 0.05 were considered statistically significant. * p < 0.05, ** p < 0.01, *** p < 0.001.

### Production of recombinant *Aa*-HSP60 protein

The cDNA construct of *Aa*-HSP60 in pET28a(+) expression vector was purchased from GenScript. The cDNA (nucleotides 529–2172 of the *Aggregatibacter actinomycetemcomitans* groEL gene) was subcloned into the EcoRI–XhoI site of pET-28a(+) vector containing a (His)_6_-tag at the amino terminus. The plasmid was transformed into *BL21 (DE3) E*. *coli* cells (Agilent Technologies), and 0.5 mM isopropyl β-D-1-thiogalactopyranoside (IPTG, Sigma) was used for protein expression. After the expression, bacterial cells were lysed (lysozyme 1 mg/mL, Sigma) and sonicated, followed by ultracentrifugation (48,000 x g for 30 min) at +4°C. For protein purification, cell lysate was collected after ultracentrifugation, and HisPur^™^ Cobalt resin (Thermo Fisher Scientific) was used for binding of the protein. The purity of the recombinant protein was analyzed with sodium dodecyl sulfate polyacrylamide gel electrophoresis (SDS-PAGE, S2 Fig).

### Chemiluminescence immunoassay

Plasma antibody levels from human and mouse were measured with a chemiluminescence immunoassay (enzyme-linked immunosorbent assay, ELISA). Antigen was dissolved in PBS (5 μg/ml) and incubated at +4°C overnight in MicroFluor plates (Thermo Scientific, Rockford, IL, USA). Automated plate washer was used to wash the plates three times with PBS containing 0.27mM ethylenediaminetetraacetic acid (EDTA). Plates were blocked with PBS-EDTA containing 0.5% fish gelatin (Fg) and incubated for one hour at room temperature. Plasma samples were diluted with 0.5% Fg-PBS-EDTA and the antibody levels were measured with alkaline phosphatase-labeled antibodies (anti-human-IgG, anti-human-IgM, anti-mouse-IgG, anti-mouse-IgM) diluted according to manufacturer specifications (Sigma-Aldrich). Chemiluminescence was detected using LumiPhos 530 (33% Lumigen) substrate, measured with luminescence multilabel counter (PerkinElmer Victor^3^V), and expressed in relative light units (RLU).

Specific binding of plasma antibodies to *Aa*-HSP60 and MAA-LDL was tested with competitive chemiluminescence immunoassay. *Aa*-HSP60 and MAA-LDL (0–100 μg/mL) were incubated with plasma samples overnight at +4°C. Incubated solutions were centrifuged at 16,000 × g at +4°C and the remaining antibody levels measured with ELISA as described above.

### Dot blot and Western blot analyses

Seven different strains of *Aa* bacteria (ATCC 29523, ATCC 43718, ATCC 33384, IDH 781, IDH 1705, CU1000, C59A, representing six serotypes, A, B, C, D, E, F, and one nonserotypeable strain X), *Porphyromonas gingivalis (Pg)*, *Tannerella forsythia (Tf)*, and *Escherichia coli* (*E*.*coli)* were examined by Dot and Western blot analysis. For dot blot analysis (S1 Raw images), bacterial samples were diluted in Tris-buffered saline (TBS) and 200 μL of bacterial suspension (0.1 mg/mL) was added to each well. Samples were loaded on pre-wetted nitrocellulose membrane on vacuum-based dot blot apparatus (BioRad). For the western blot analysis (S1 Raw images), the same bacterial samples were used and 20 μg of bacterial protein from each strain was loaded. 15 μg of MAA-BSA and BSA as well as 0.2 μg of *Aa*-HSP60 protein were also used in SDS-PAGE. After the proteins were separated, the nitrocellulose membrane was used for blotting (BioRad). Membranes were blocked in 5% BSA-TBS buffer overnight at +4°C. All plasma samples were combined and diluted at 1:1000 in 5% BSA-0.05%Tween 20 -TBS buffer (5%-BSA-TBS-T). The samples were incubated for 1 h at room temperature. Goat-antimouse-IgG-IRDye 800 (0.25 μg/mL) was used as the secondary antibody and incubated for 1 h at room temperature. Dot blot and Western blots were visualized with Odyssey IR imager and Image Studio^™^ Software (LI-COR Biosciences).

### Flow cytometry analysis

Human Jurkat T cells were grown at +37°C with 5% CO_2_ in RPMI-1640 (Sigma) containing 10% fetal bovine serum (Thermo Fisher Scientific Inc.), 100 U/mL penicillin and 100 μg/mL streptomycin (Sigma), 10 mM Hepes, 2 mM L-glutamine, and 1 mM sodium pyruvate. Apoptosis was induced by starving in serum-free RPMI-1640 for 24 hours. Cells (5 × 10^5^) were washed with 0.1% BSA in PBS and centrifuged at 1,800 × g for 5 minutes at +4°C. The apoptotic cells were stained with SYTOX^®^AADvanced^™^ Dead Cell Stain Kit (Life Technologies). Jurkat T cells were incubated with mouse plasma (1:100) before and after immunization and with mouse plasma (1:100) pre-mixed with *Aa*-HSP60 (100 μg/mL) by shaking at +4°C for 45 minutes. Goat anti-mouse IgG (H+L) or IgM (μ chain) Alexa Fluor 488 (Invitrogen) was used, respectively, as secondary antibody at a concentration of 0.25 μg/mL. The washing was repeated after antibody incubation. Binding of the IgM and IgG antibodies to apoptotic cells was analyzed with BD Accuri C6 Plus instrument (BD Biosciences) and FlowJo V10.1 software (FlowJo LLC).

### Statistical analyses

Statistical analyses were carried out with IBM SPSS statistic 25 software. In human study Mann-Whitney U test was used to compare the variables in different groups and Wilcoxon signed-rank test to compare the variables within the same groups. T-test was used in mouse study. P-values less than 0.05 were considered significant (* p < 0.05, ** p < 0.01, *** p < 0.001).

## Results

### Human umbilical cord blood contains natural IgM to *Aa*-HSP60 and MAA-LDL

Human umbilical cord blood plasma and maternal plasma were tested for IgG and IgM antibodies binding to MAA-LDL, *Aa*-HSP60 and Fg-PBS (Fig 1). The IgG antibody binding patterns of mothers and neonates were very similar (Fig 1A). Not much difference was detected between the IgG antibody levels of mothers and neonates, indicating placental transfer of maternal IgG antibodies to the fetus, an important mechanism that provides protection to the infant.

Neonates did have significant IgM levels to *Aa*-HSP60 and MAA-LDL when compared to Fg-PBS background controls (Fig 1B), suggesting that the IgM antibodies to *Aa*-HSP60 and MAA-LDL are already being produced by neonates before birth. *Aa*-HSP60 could compete effectively with the IgM binding to MAA-LDL (Fig 1C), suggesting that *Aa*-HSP60 may share molecular mimicry with MAA epitopes in OxLDL. However, the competitive binding of plasma IgM antibodies to *Aa*-HSP60 could not be seen by using MAA-LDL as a competitor (Fig 1D).

### Prominent IgG and IgM, but not IgA, immune responses to *Aa*-HSP60

**Fig 2:**
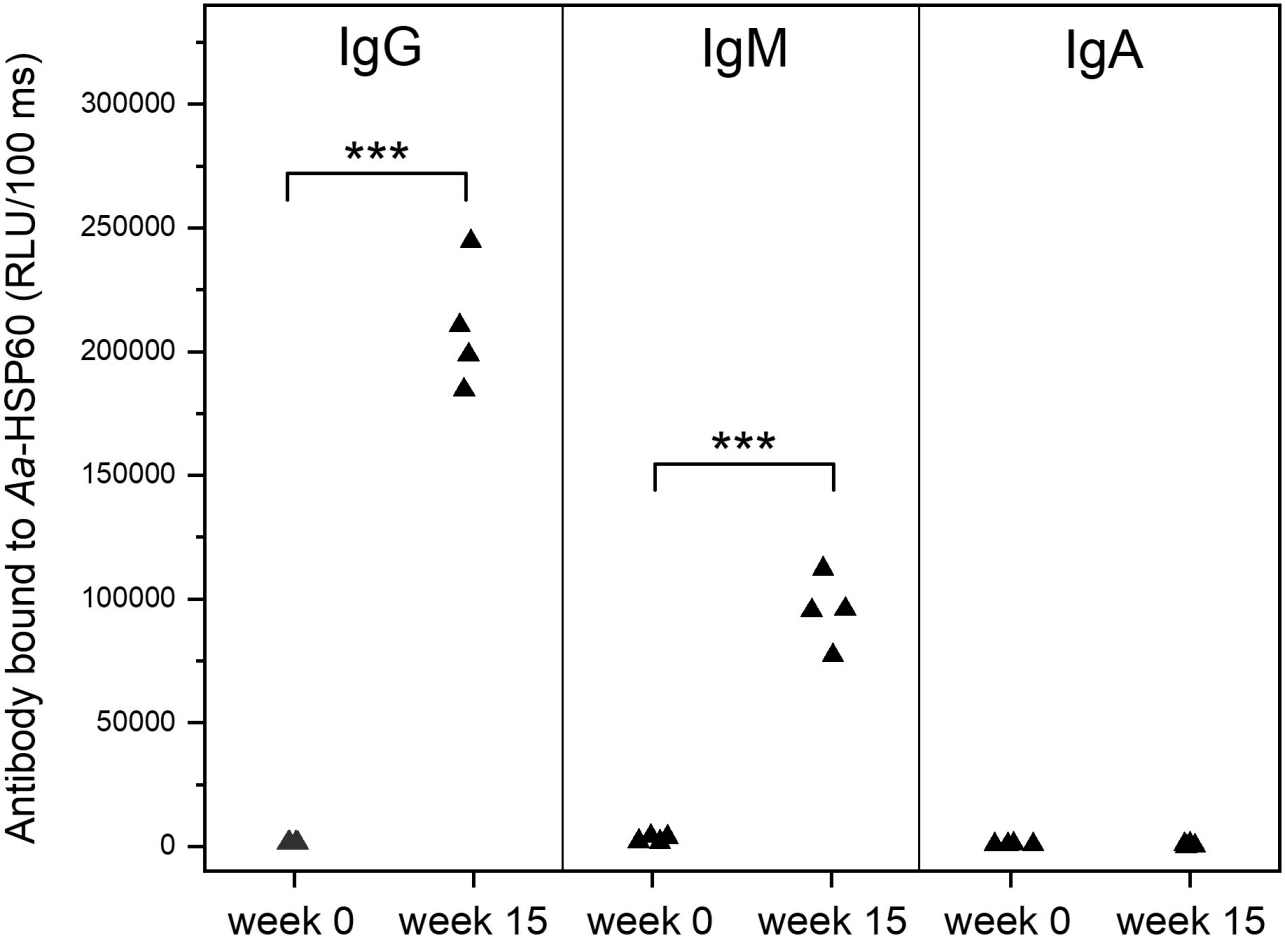
Mouse plasma IgG, IgM, and IgA antibody levels to *Aa*-HSP60 before (week 0) and after (week 15) immunization. The mouse (n = 4) antibody binding is expressed in relative light units measured in 100 milliseconds (RLU/100ms). Antibody levels are shown as box-plots, representing 25%, 50% and 75% of the distribution, and whiskers representing 10% and 90% distribution of the values. The cross represents the maximum and minimum values and the solid diamonds are the mean values.

C57BL/6 female mice were immunized with the purified recombinant heat shock protein 60 of *Aggregatibacter actinomycetecomitans* (*Aa*-HSP60). Strong IgG and IgM immune responses to *Aa*-HSP60 were detected in all mice after immunization. IgG and IgM levels to *Aa*-HSP60 were remarkably increased at week 15 when compared to the antibody levels before immunization (Fig 2). However, no IgA response to immunization of *Aa*-HSP60 was observed throughout the experimental period (Fig 2).

### Plasma IgG and IgM antibody levels to MAA-LDL increased after *Aa*-HSP60 immunization

**Fig 3:**
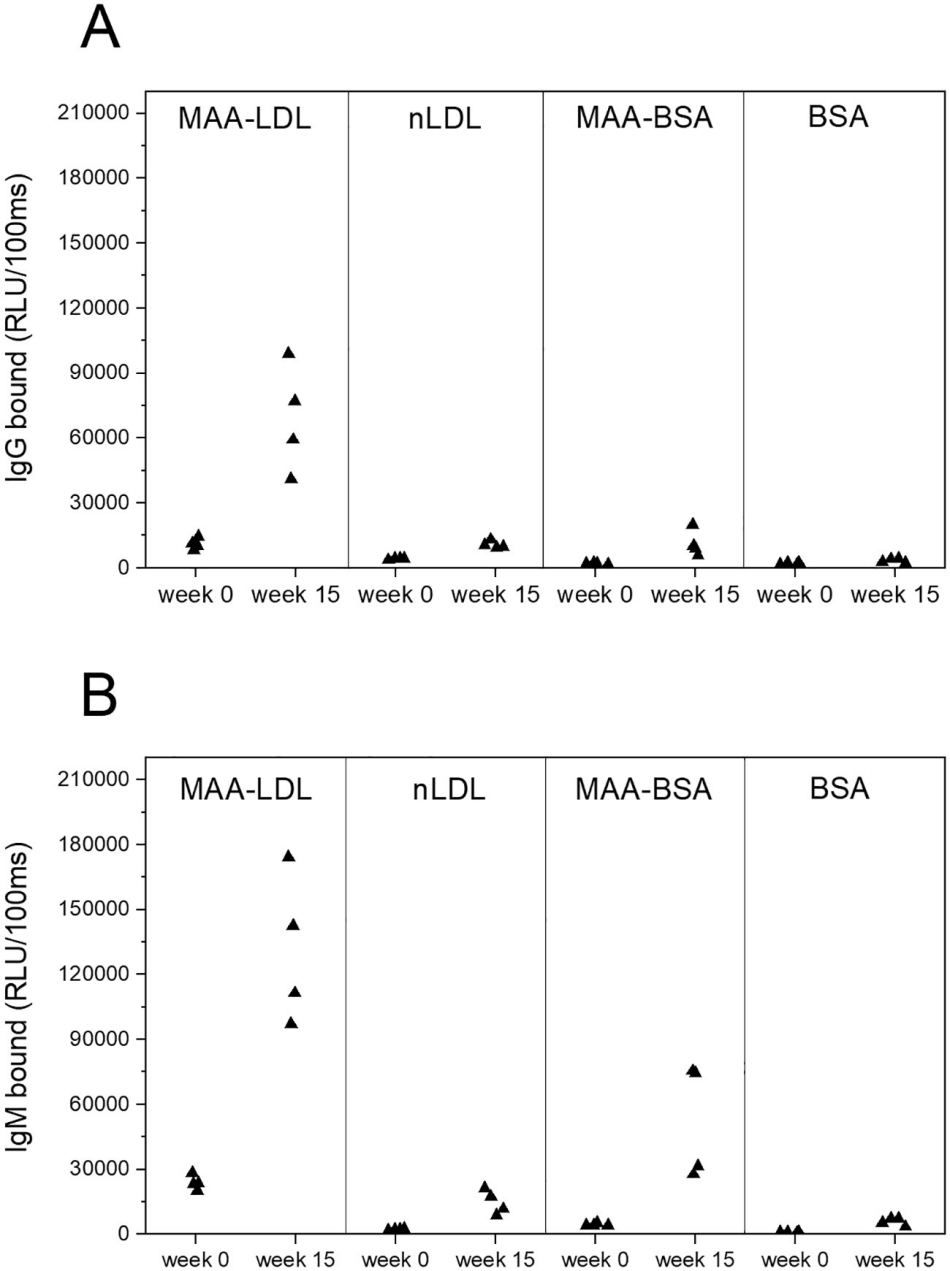
Mouse plasma IgG and IgM antibody levels to MAA-LDL and MAA-BSA before (week 0) and after (week 15) immunization. Unmodified natural LDL (nLDL) and bovine serum albumin (BSA) were used as controls. The mouse (n = 4) antibody binding is expressed in relative light units measured in 100 milliseconds (RLU/100ms). Antibody levels are shown as box-plots, representing 25%, 50% and 75% of the distribution, and whiskers representing 10% and 90% distribution of the values. The cross represents the maximum and minimum values and the solid diamonds are the mean values.

To investigate the cross-reactivity of antibodies to *Aa*-HSP60 and MAA-LDL, plasma from mice immunized with AaHSP60 was also tested for binding to MAA-LDL and MAA-BSA. Unmodified natural LDL (nLDL) and bovine serum albumin (BSA) were used as controls. The binding results of the IgG and IgM antibodies are presented in Fig 3. The antibody levels against MAA-LDL were remarkably increased at week 15 when compared to the levels before immunization (Fig 3A and 3B). The IgM antibody level to MAA-BSA was also notably elevated (Fig 3B). Both IgG and IgM antibody levels to nLDL and BSA remained low throughout the immunization period.

### Competitive chemiluminescence immunoassay shows specific binding of IgG and IgM antibodies to *Aa*-HSP60

**Fig 4:**
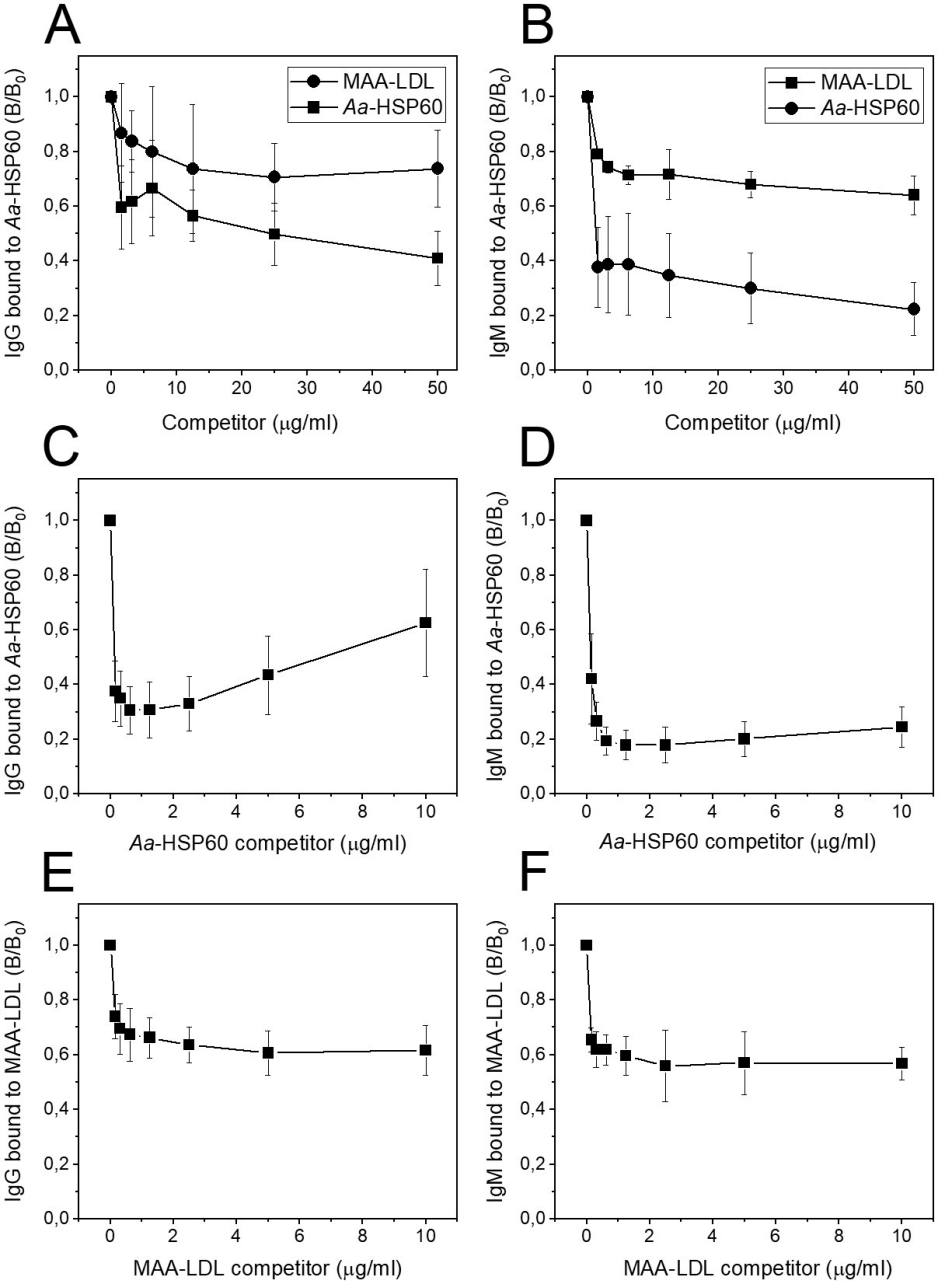
Competitive immunoassay of mouse plasma IgG and IgM binding to fixed Aa-HSP60 or MAA-LDL after incubation with competitor. The binding specificity of IgG and IgM class antibodies are presented as ratio of binding with (B) or without competitor (B_0_). The values are average values of four mice (± standard deviation). (A) IgG binding to fixed *Aa*-HSP60 after competed with MAA-LDL or *Aa*-HSP60. (B) IgM binding to fixed *Aa*-HSP60 after competed with MAA-LDL or *Aa*-HSP60. (C) IgG binding to fixed *Aa*-HSP60 after competed with *Aa*-HSP60. (D) IgM binding to fixed *Aa*-HSP60 after competed with *Aa*-HSP60. (E) IgG binding to fixed MAA-LDL after competed with MAA-LDL. (F) IgM binding to fixed MAA-LDL after competed with MAA-LDL.

The specific binding to *Aa*-HSP60 and cross-reactivity with MAA-LDL of mouse plasma antibodies was examined by outcompeting antibodies with increased levels of *Aa*-HSP60 and MAA-LDL as competitors (Fig 4). *Aa*-HSP60 competed effectively with the whole plasma antibody binding, especially IgM, to *Aa*-HSP60 whereas MAA-LDL competed partially with the binding (Fig 4A and 4B). Almost 70% of the IgG class antibody binding to *Aa*-HSP60 was outcompeted by liquid *Aa*-HSP60 competitor at less than 1 μg/mL concentrations (Fig 4C). About 80% of the IgM antibody binding to *Aa*-HSP60 was also outcompeted by *Aa*-HSP60 at very low concentrations (Fig 4D). The data suggest that both IgG and IgM class of antibodies are generated specifically towards *Aa*-HSP60 with immunization. IgG and IgM specificity to MAA-LDL was also tested (Fig 4E and 4F). Around 40 percent of both antibodies present in plasma were outcompeted by liquid MAA-LDL competitor, implying that certain antibodies produced after *Aa*-HSP60 immunization recognized MAA-LDL but could not be well competed by MAA-LDL. Cross-reaction of the antibodies between *Aa*-HSP60 and MAA-LDL could also be visualized in Fig 4A and 4B. Approximately 30 percent of the antibodies binding to fixed *Aa*-HSP60 were outcompeted by liquid MAA-LDL competitor, implying that MAA-LDL cross-reacts with antibodies against *Aa*-HSP60.

### Immunized mouse plasma antibodies recognized *Aa* bacteria in Dot and Western blot analysis

**Fig 5:**
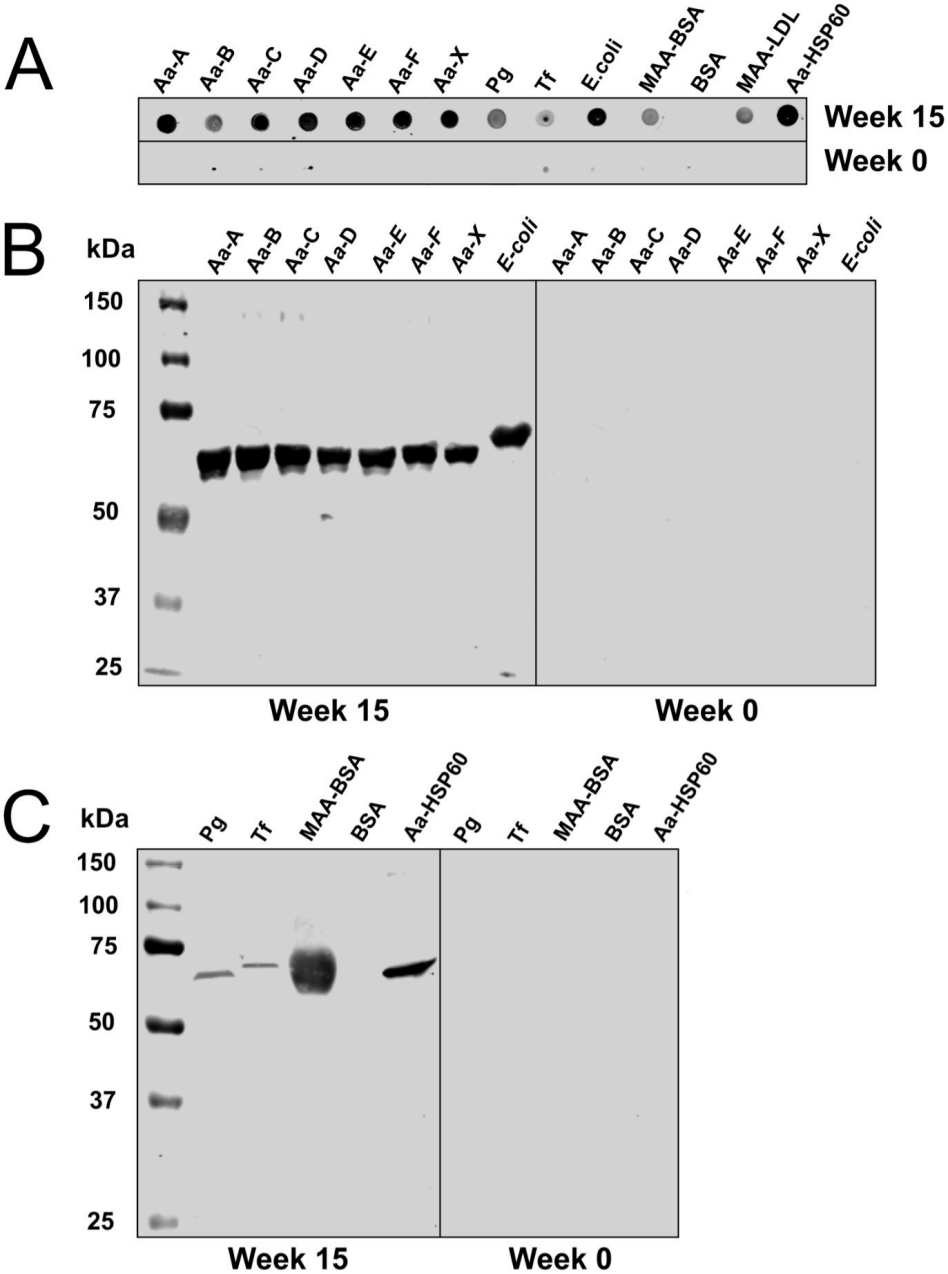
Dot blot and Western blot assays. Seven different strains of *Aa*-bacteria (*Aa*-A, *Aa*-B, *Aa*-C, *Aa*-D, *Aa*-E, *Aa*-F, *Aa*-X), *Pg*, *Tf* and *E*.*coli* bacteria are presented before (week 0) and after (week 15) immunization, together with MAA-BSA, BSA, and *Aa*-HSP60. Dot blot results are presented in Fig 5A. Western blot results are presented in Figs 5B and 5C. Samples were separated by SDS-PAGE and blotted by Western Blotting. Molecular weight marker in kilodaltons (kDa) is shown on the left. Fragments of the same original image were spliced together to re-order lanes to remove irrelevant lanes.

To test whether mouse plasma has cross-reactivity with pathogenic microbes after *Aa*-HSP60 immunization, the antibody binding to *Aggregatibacter actinomycetemcomitans (Aa)*, *Porphyromonas gingivalis (Pg)*, *Tannerella forsythia (Tf)*, *and Escherichia coli* (*E*.*coli)* was examined by Dot and Western blot analysis. Plasma samples taken before immunization were used as controls. Dot blot showed strong plasma binding with all serotypes of *Aa*, *E*.*coli*, and recombinant *Aa*-HSP (Fig 5A), whereas mild binding was observed from *Pg*, *Tf*, MAA-BSA and MAA-LDL (Fig 5A). No binding was detected from BSA (Fig 5A). All *Aa* bacteria were strongly recognized on Western blot with a protein band at around 60–65 kDa, corresponding to the predicted size of HSP60 (Fig 5B). A clear band was also visualized, respectively, with *Pg*, *Tf*, and *E*. *coli*. Correct-sized bands were also detected in both MAA-BSA- and *Aa*-HSP60-loaded lanes (Fig 5C). BSA showed no visible bands. Plasma samples before immunization (week 0) did not recognize any proteins in any of the tested bacterial lysates or protein controls (Fig 5B and 5C).

### Plasma IgM binding to apoptotic cells can be competed by *Aa*-HSP60 in immunized mice

**Fig 6:**
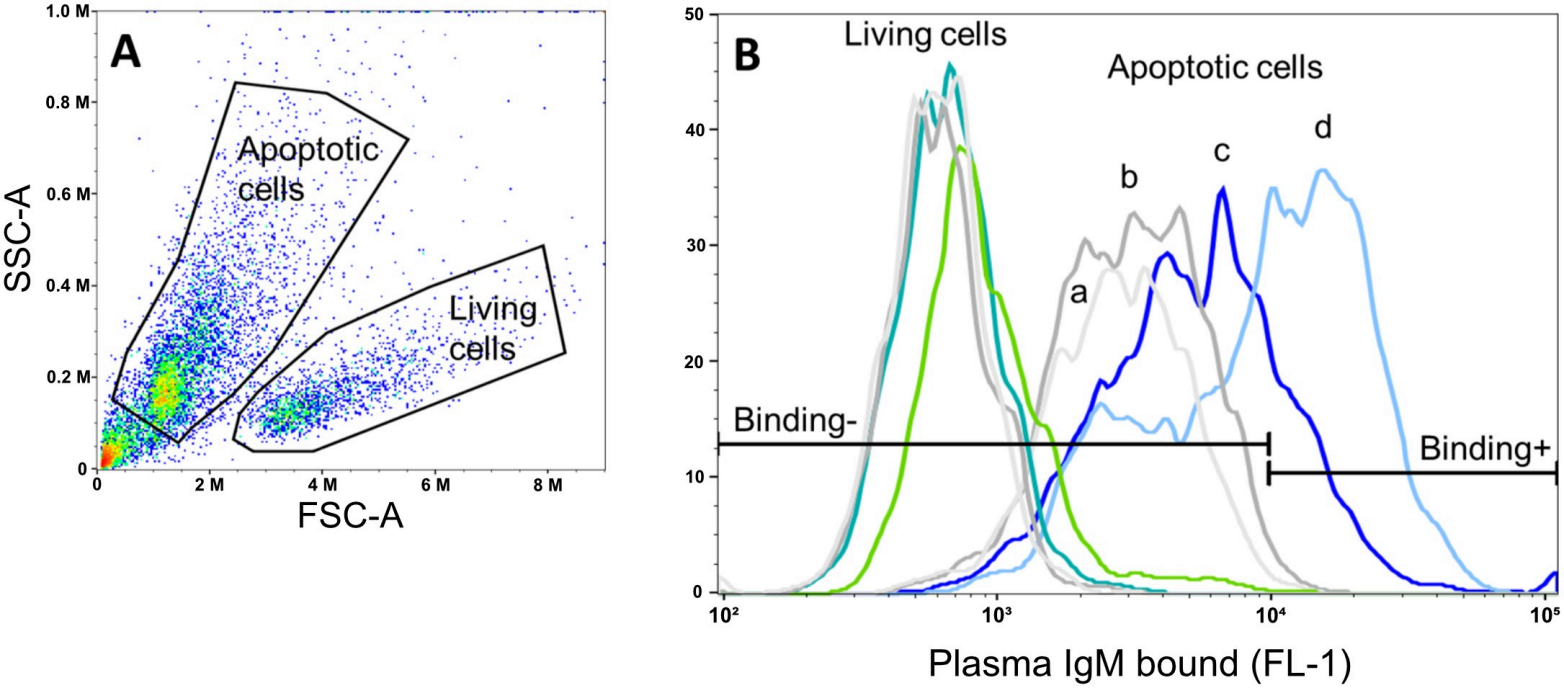
Flow cytometry analysis of mouse plasma IgM binding to apoptotic cells after *Aa*-HSP60 immunization. (A) Apoptotic and living Jurkat cells were gated based on the staining with SYTOX^®^AADvanced Dead Cell Staining Kit. (B) Mouse plasma (1:100) IgM binding to living and apoptotic cells. Apoptotic cell panel: a: apoptotic cells alone; b: secondary antibody control; c: IgM binding to apoptotic cells with pre-mixed *Aa*-HSP60 (100 μg/mL); d: IgM binding to apoptotic cells without pre-mixed *Aa*-HSP60. Living cell panel: light grey: living cells alone; grey: secondary antibody control; blue: IgM binding to living cells with pre-mixed *Aa*-HSP60; green: IgM binding to living cells without pre-mixed *Aa*-HSP60.

To test if mouse plasma IgM or IgG bound to apoptotic cells after *Aa*-HSP60 immunization, the binding to apoptotic Jurkat T cells was analyzed by flow cytometry (Fig 6). Mouse plasma without immunization was taken as control. Apoptotic and living cells were populated with SYTOX^®^AADvanced^™^ Dead Cell Stain Kit (Fig 6A). No plasma IgM binding to living Jurkat cells was observed whereas the binding to apoptotic cells was very strong (Fig 6B). The IgM binding to apoptotic cells was also competed out by recombinant *Aa*-HSP60 at a concentration of 100 μg/mL (Fig 6B). Mouse plasma IgM binding to apoptotic cells without immunization showed weak competetion by *Aa*-HSP60 (S3 Fig). Flow cytometry analysis also revealed that neither of the IgG antibodies from immunized or non-immunized mice bound to apoptotic cells (S4 Fig).

## Discussion

The present study demonstrates the recognition of *Aa*-HSP60 and MAA-LDL by natural antibodies from neonates and reveals the existence of cross-reactivity between *Aa*-HSP60 and MAA-LDL. Mouse study confirms that humoral immune response against *Aa*-HSP60 also generates antibodies cross-reacting with oxidized LDL molecule (MAA-LDL). *Aa*-HSP60 immunization provokes IgG and IgM antibody responses recognizing key periodontal pathogens without changing IgA antibody levels against *Aa*-HSP60 in C57BL/6J mice.

MAA adduction results from an ineffective clearance of reactive oxygen species. When exposed to oxidative stress, cell walls may rupture and membrane lipids oxidize into malondialdehyde (MDA) and break down spontaneously, forming acetaldehyde (AA). Both MDA and AA are highly reactive and are able to modify ε-amino groups of lysine residues of proteins to produce stable malondialdehyde-acetaldehyde (MAA) protein adducts [3,18]. The heat shock proteins are highly conserved proteins with important functions in protein homeostasis and cell signaling. HSP60 is a “danger signal” to the immune system and is very immunogenic [19]. MAA epitopes and HSP60 are both elevated under similar stressed conditions, and antibodies to these structures are both associated with atherosclerosis [13,16].

We have previously cloned a natural mouse monoclonal IgM antibody against MAA epitope, which cross-reacts with HSP60 of the *Aa* bacteria [17], indicating molecular mimicry between MAA and HSP60. In this study, we show that immunization with *Aa*-HSP60 in mice induced remarkable IgM and IgG responses to MAA-LDL. The data provide further evidence showing the existence of molecular mimicry between the *Aa*-HSP60 and MAA epitope that can be recognized via natural antibodies or antibodies from adaptive immunity. It is now clear that both innate and adaptive immune responses are intimately related to atherogenesis. Both IgG and IgM antibodies binding to fixed *Aa*-HSP in neonates and in immunized mice were effectively outcompeted by *Aa*-HSP, but partially competed by MAA-LDL, implying that the induced antibodies are not ideally bound to MAA-LDL or are less specific to MAA-LDL. Oxidation specific epitopes, including MAA-epitopes, have been shown to be a major target of natural IgM antibodies in mice and humans. Studies in human and animal models have shown that plasma levels of IgM antibodies to oxidized LDL are inversely correlated with atherosclerosis [6]. IgM antibodies from the umbilical cord blood originate from the developing fetus as a germline-encoded repertoire of natural IgM against the shared epitopes of MAA and HSP60. The data imply that clearance of oxidized proteins plays an important physiological role in protection from harmful changes to proteins. The mouse plasma IgM, but not IgG, in immunized mice recognizes apoptotic Jurkat cells. The result is consistent with the opinion that IgM antibodies to MAA-LDL may have a very important role in atheroprotection by inhibiting the macrophages uptake of the oxidized LDL, and by enhancing apoptotic cell clearance [3,4]. Little is known about the role of IgM antibodies to HSP60 in the development of atherosclerosis. Our study is the first report demonstrating human natural IgM to *Aa*-HSP60 existing at birth and cross-reacting with MAA-LDL. It can be speculated that IgM antibodies to *Aa*-HSP60 might be atheroprotective in newborns as they cross-react with MAA-LDL. The exact reason why *Aa*-HSP60 is recognized by the natural repertoire of IgM is unknown. Further investigations are needed to address the comprehensive physiological questions, e.g. why natural IgM in the fetus recognizes the microbial pathological virulence factor before birth and what the exact role of natural IgM to *Aa*-HSP60 plays in atherogenesis.

It is unknown what kinds of structures shared by the major protein component (ApoB-100) of MAA-LDL and HSP60 lead to the similar immune responses. Sequence alignment revealed only one region of 21 amino acids (amino acid 118–137 in Aa-HSP60 and amino acid 356–367 in ApoB100) at the N-terminus of both proteins sharing about 43% identities. Immunization with ApoB-100 (amino acids 661–680) and HSP60 (amino acids 153–163) peptide antigens together has been shown to exert synergetic atheroprotective effect [20]. A multivalent vaccine combining immunogenic epitopes of HSP60, ApoB-100, and β2 glycoprotein I in a chimeric protein has also been suggested as a potential candidate for modulation and reduction of atherosclerosis [21]. Nearly all naturally occurring antibody epitopes studied are composed of amino acids that are sporadic in the primary sequence but brought together in space by protein folding in the tertiary structure [22]. Approximately 50 variable amino acids build up the potential binding area of an antibody [23], and only about one-third of them physically contact a particular epitope. These contact residues define the structural paratope. Changes in amino acids in both the epitope of an antigen and paratope of an antibody lead to a change in spatial conformation of the binding region and affect the binding reaction. A particular epitope can be recognized by two different paratopes with no sequence similarity [22]. Therefore, it can be speculated that optimal 3D structure might be more preferred than primary amino acid sequence for molecular mimicry between the two molecules. However, it is not clear which amino acids were preferentially used by *Aa*-HSP60 to make the mimic to the MAA epitope. Solving these questions in future by structure biology methods will reveal the impact of *Aa*-HSP60 on the regulation of the immune system involved in the development of atherosclerosis.

Periodontitis has been associated with atherosclerosis [24]. The most studied pathogenic microbes are gram-negative bacteria, such as *Aggregatibacter actinomycetemcomitans (Aa)* and *Porphyromonas gingivalis (Pg)*. Previous studies suggest that vaccination against virulence factors of the oral pathogens may confer disease resistance [4,25]. Similarly, OxLDL-based vaccinations have been developed to modulate the progression of atherosclerosis [26]. The exact mechanism between atherosclerosis and periodontitis remains unknown but the cross-reaction of antibodies provides a new insight into the pathogenesis of these diseases. We have previously cloned mouse monoclonal IgM antibodies to MDA- and MAA-LDL [17,27]. They cross-react, respectively, with a hemagglutinin domain of gingipain protease of *Pg* (Rgp44) and *Aa*-HSP60. We have also reported that immunization with Rgp44 reduces atherosclerosis in LDL receptor-deficient (LDLR^-/-^) mice and that increased IgM levels to MAA-LDL may contribute to atheroprotection [4]. Therefore, *Aa*-HSP60-induced antibody response to MAA-LDL may cross-react with virulence factors, such as Rgp44, from *Pg* bacteria.

We show in the current study that antibodies from mouse plasma recognized *Aa*, *Pg*, and *Tf* periodontal pathogens after immunization. This suggests that the immunogenic property or the ‘danger signal’ of *Aa*-HSP60 provoked strong IgG and IgM antibody responses, which might be beneficial for elimination of invading pathogens possessing epitopes shared by HSP60 and MAA-LDL. It is very interesting to notice the strong plasma antibody binding to *Escherichia coli (E*. *coli*) bacteria. According to SmartBLAST search, chaperonin GroEL of *E*. *coli* is found to be the closest relative to *Aa*-HSP60 among the matched sequences in the phylogentic tree, which may explain the notable binding. Post-translational modifications (PTMs) of proteins may contribute significantly to the mobility shift of HSP60 in our tested bacteria as remarkable differences in expression and occupancy of PTMs sites under different growth conditions have been found in bacteria [28].

In this study, we also expanded our interest to adaptive immune responses of mice to determine possible cross-reactions and similarities to innate immunity. We demonstrated that *Aa*-HSP60 immunization significantly increased IgG and IgM antibody titer to MAA and HSP60, both of which are strongly associated with the progression of atherosclerosis. However, IgA antibody levels against *Aa*-HSP60 and MAA-LDL remained unchanged. It has been shown that increased titers of different isotypes of anti-MAA antibodies (IgG, IgM, IgA) predict atherosclerosis progression and cardiovascular events [16]. Also in acute myocardial infarction (AMI), anti-MAA antibody titers are increased and correlate with the severity of the disease [9]. In the isotype evaluation, there is a pathological association between IgG antibody titer and AMI [9]. IgG and IgM antibody titers to MAA decrease after AMI, which is hypothesized to be due to the release of MAA proteins and activation of complement system [9]. Similarly, AMI leads to release of HSP60 protein, suppressing the humoral immune response to HSP60 via immune complex activation [29].

We used standard C57BL/6J laboratory mice that are not ideal for developing atherosclerosis compared to the apoE^-/-^ and the LDLR^-/-^ mouse models [30], making it difficult to determine whether the *Aa*-HSP60 immunization is atheroprotective or pro-atherogenic. Our objective in this study was not to determine the progression of atherosclerosis in mice but to study the antibody responses after *Aa*-HSP60 immunization. It has been reported that administration route of HSP60 immunization could affect the progression of atherosclerosis [31]. The current understanding is that HSP60 oral administration protects from atherosclerosis due to the HSP60-induced mucosal immune tolerance, whereas subcutaneous injection promotes atherosclerosis progression [12,31–33]. Oral administrations of HSP60 increase MSDCs (myeloid derived suppressor cells) that suppress the progression of atherosclerosis [31]. IgA is the dominant immunoglobulin for mucosal defense and the second most abundant found in the circulation. Great differences have been observed between the glycosylation of saliva and plasma IgA. It is suggested that salivary IgA produced locally by plasma B cells in the glandular stroma differs from the IgA in the circulation which is presumably produced by circulating plasma cells [34]. It is unknown why plasma IgA antibodies to *Aa*-HSP60 and MAA-LDL remained unchanged in this study after immunization. Oral administration might be a better way to stimulate IgA production compared to intraperitoneal injection. A distinct regulatory mechanism may be responsible for the phenomenon.

In summary, this study provides the first evidence that natural IgM antibodies to *Aa*-HSP60 exist in neonates even before birth. *Aa*-HSP60 shares molecular mimicry with oxidized MAA epitopes, by which the spatial confirmation may be preferred to induce antibody cross-reaction. The study gives new insights into understanding how the immune system responds to virulence factors of periodontal pathogens. It may provide an opportunity for paving the way towards an immune-modulatory strategy to restrain inflammatory diseases such as atherosclerosis.

## Supporting information

S1 Fig Experimental timeline for immunizations and blood sample collections. (TIFF)

S2 Fig SDS-PAGE of the purification of the recombinant *Aa*-HSP60. MW was indicated on the left side. Lane 1: protein markers; Lane 2: crude cell lysate; Lane 3: cell pellet; Lane 4: unbound fraction; Lane 5–7: washing fractions; Lane 8–10: elution fractions containing the purified *Aa*-HSP60.
(TIFF)

S3 Fig Flow cytometry analysis of mouse plasma IgM binding to apoptotic cells before *Aa*-HSP60 immunization. The IgM binding to apoptotic cells was tested with (blue) or without (light blue) pre-mixed *Aa*-HSP60 as a competitor (100 μg/mL). A weak competition was observed from plasma IgM binding to apoptotic cells in the presence of pre-mixed *Aa*-HSP60.
(TIFF)

S4 Fig Flow cytometry analysis of mouse plasma IgG binding to apoptotic cells with and without *Aa*-HSP60 immunization. No plasma IgG binding to apoptotic cells was observed in immunized and pre-immunized mice.
(TIFF)

S1 Raw images Raw data from Dot blot and Western blot analysis. (PDF)
