# Supplementary figures and images for: Humoral immune response to heat shock protein 60 of Aggregatibacter actinomycetemcomitans and cross-reactivity with malondialdehyde acetaldehyde-modified LDL
Source: PLoS One. 2020 Mar 25;15(3):e0230682. doi: 10.1371/journal.pone.0230682 (PMC7094845; doi:10.1371/journal.pone.0230682)

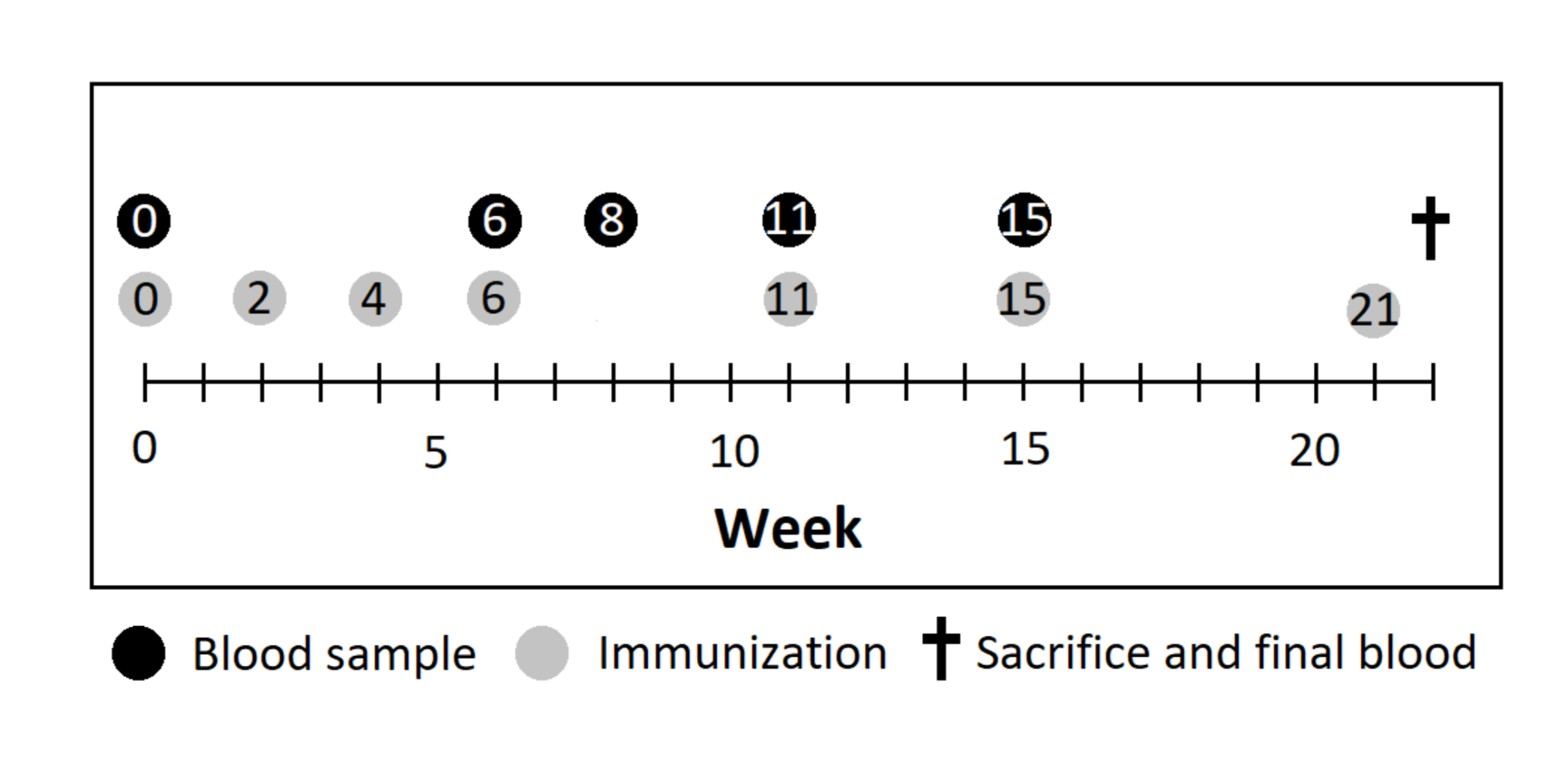

Supplement: S1 Fig — (TIFF) [file pone.0230682.s001.tiff]

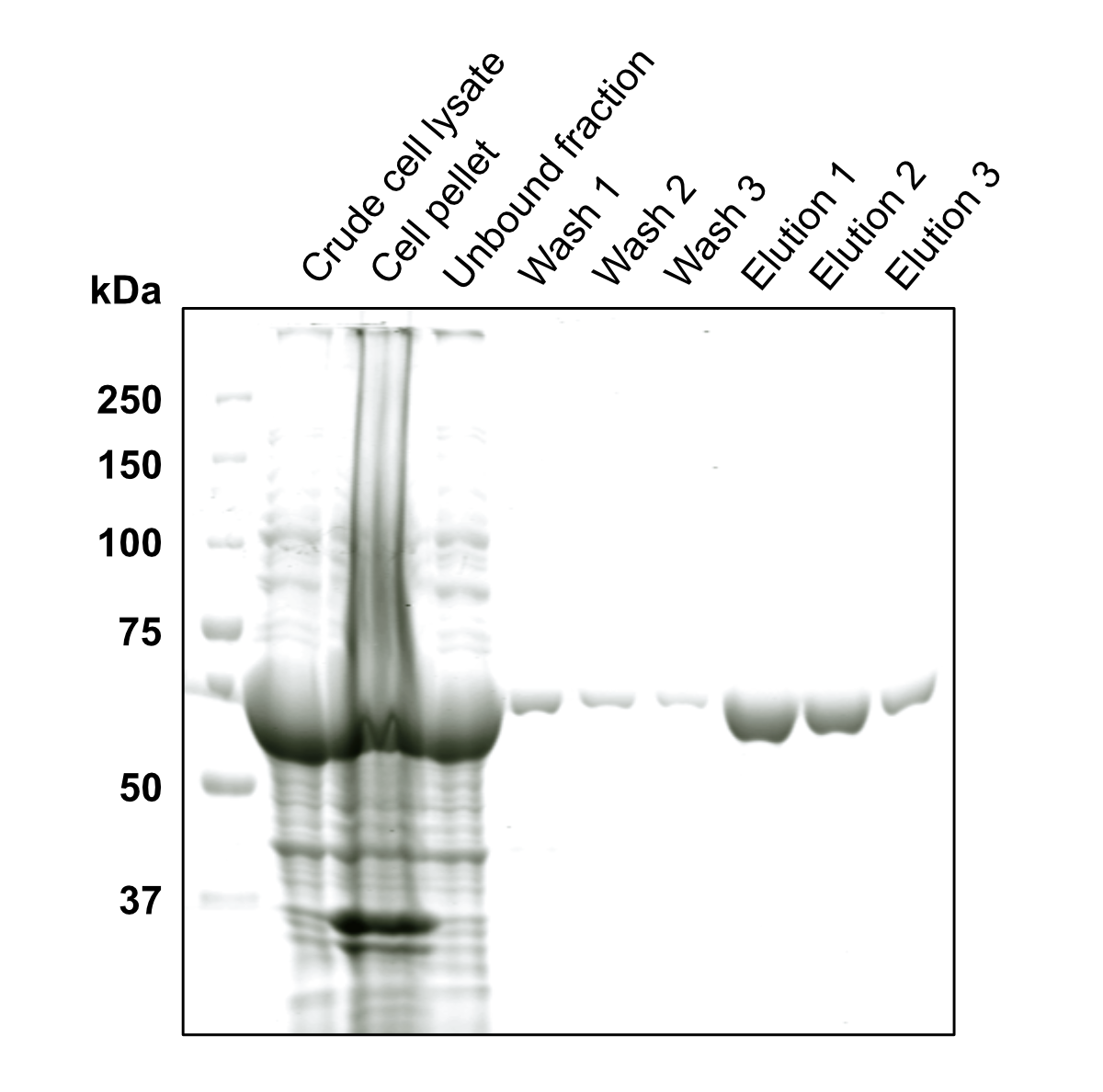

Supplement: S2 Fig — MW was indicated on the left side. Lane 1: protein markers; Lane 2: crude cell lysate; Lane 3: cell pellet; Lane 4: unbound fraction; Lane 5–7: washing fractions; Lane 8–10: elution fractions containing the purified Aa-HSP60. (TIFF) [file pone.0230682.s002.tiff]

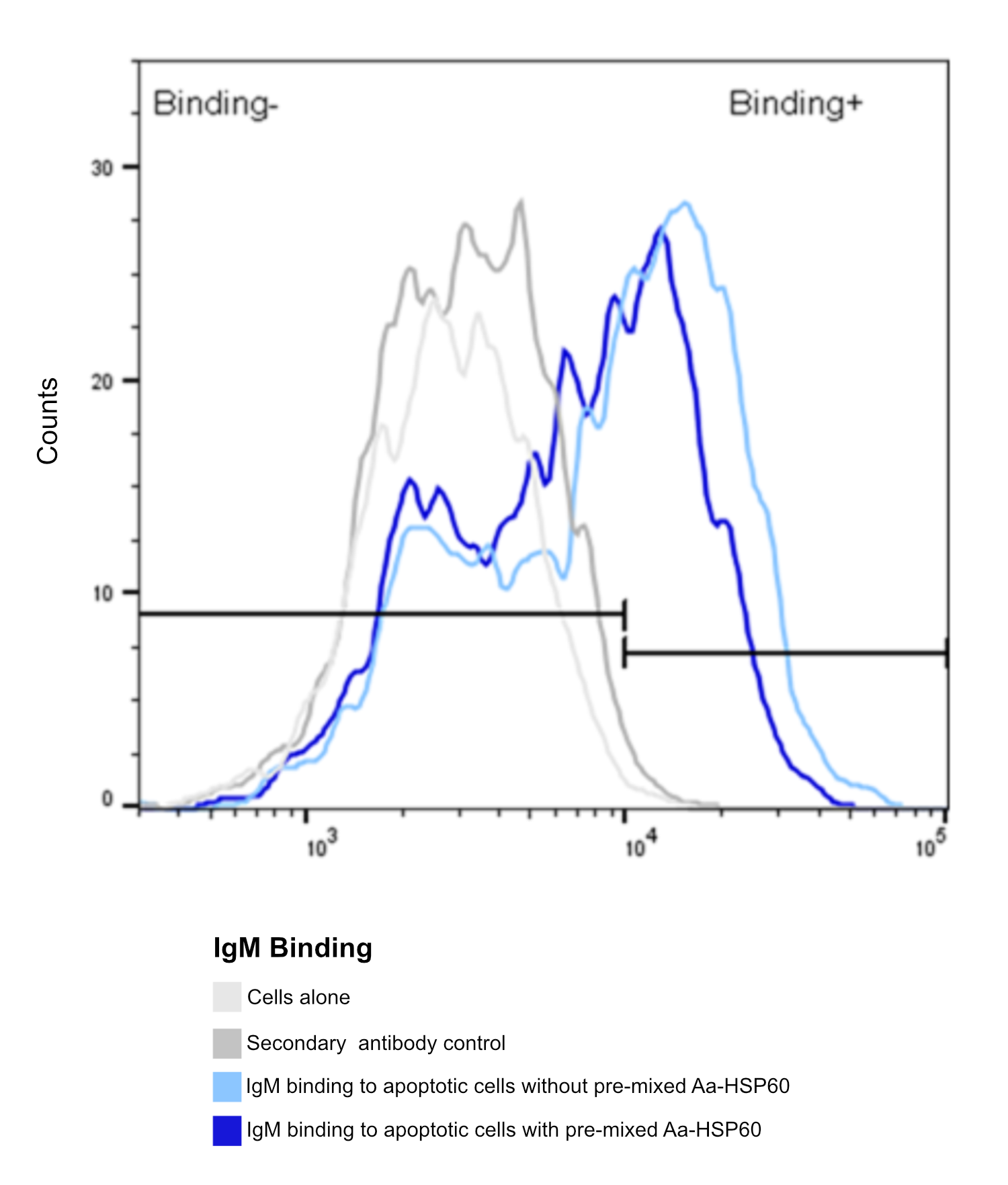

Supplement: S3 Fig — The IgM binding to apoptotic cells was tested with (blue) or without (light blue) pre-mixed Aa-HSP60 as a competitor (100 μg/mL). A weak competition was observed from plasma IgM binding to apoptotic cells in the presence of pre-mixed Aa-HSP60. (TIFF) [file pone.0230682.s003.tiff]

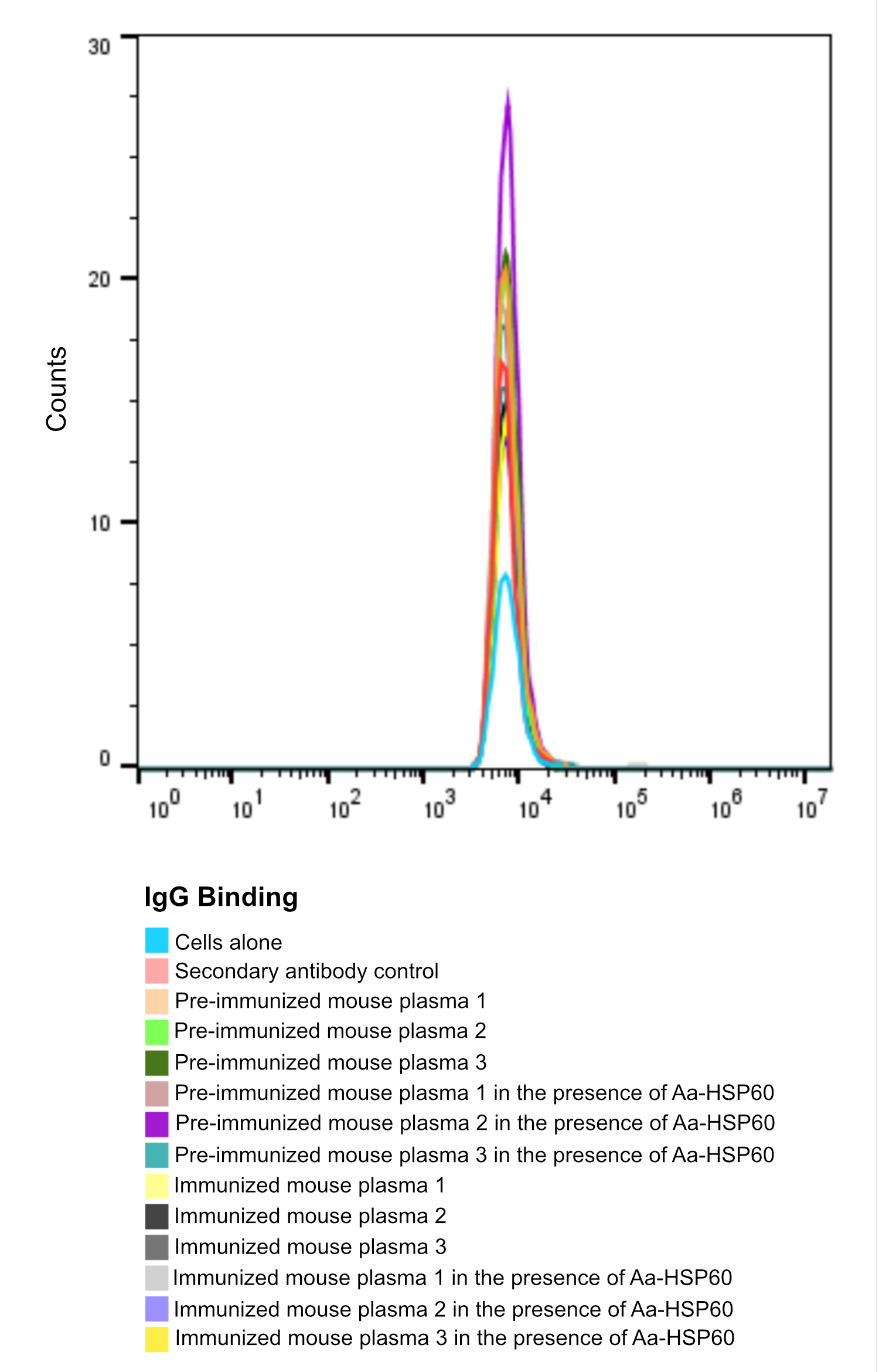

Supplement: S4 Fig — No plasma IgG binding to apoptotic cells was observed in immunized and pre-immunized mice. (TIFF) [file pone.0230682.s004.tiff]
