# Supplementary material for: Humoral immune response to heat shock protein 60 of Aggregatibacter actinomycetemcomitans and cross-reactivity with malondialdehyde acetaldehyde-modified LDL
Source: PLoS One. 2020 Mar 25;15(3):e0230682. doi: 10.1371/journal.pone.0230682 (PMC7094845; doi:10.1371/journal.pone.0230682)
Supplement: S1 Raw images — (PDF) [file pone.0230682.s005.pdf]

Dot Blot

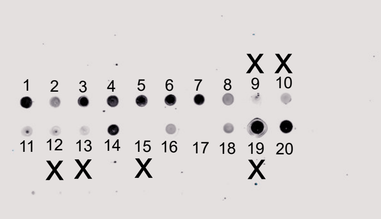

X = Not used in the study
1 - 7 = *A.a* bacteria
8 - 10 = *P.g* bacteria
11 - 13 = *T.f* bacteria
14 = *E.coli*
15 and 17 = BSA
16 = MAA-BSA
18 = MAA-LDL
19 and 20 = *Aa*-HSP60

Western Blot 1

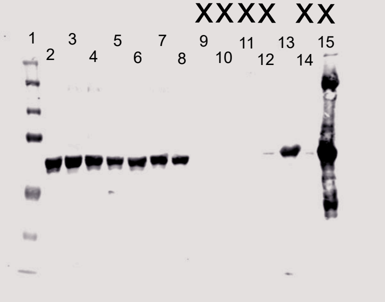

X = Not used in the study
1 = Molecular weight marker
2 - 8 = *A.a* bacteria
9 - 11 = Empty well
12 = *P.g* bacteria
13 = *E.coli*
14 = *T.f* bacteria
15 = *Aa*-HSP60

Western Blot 2

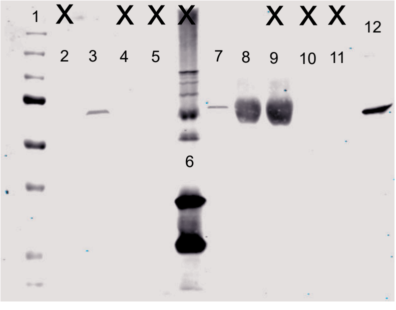

X = Not used in the study
1 = Molecular weight marker
2, 4, 5, 10, 11 = Empty well
3 = *P.g* bacteria
6 = *Pseudomonas aeuruginosa*
7 = *T.f* bacteria
8 and 9 = MAA-BSA
12 = *Aa*-HSP60

Dot Blot (week 0)

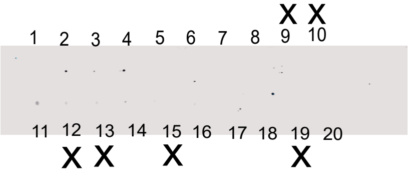

X = Not used in the study
1 - 7 = *A.a* bacteria
8 - 10 = *P.g* bacteria
11 - 13 = *T.f* bacteria
14 = *E.coli*
15 and 17 = BSA
16 = MAA-BSA
18 = MAA-LDL
19 and 20 = *Aa*-HSP60

Western Blot 1 (week 0)

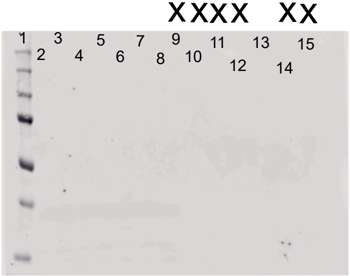

X = Not used in the study
1 = Molecular weight marker
2 - 8 = *A.a* bacteria
9 - 11 = Empty well
12 = *P.g* bacteria
13 = *E.coli*
14 = *T.f* bacteria
15 = *Aa*-HSP60

Western Blot 2 (week 0)

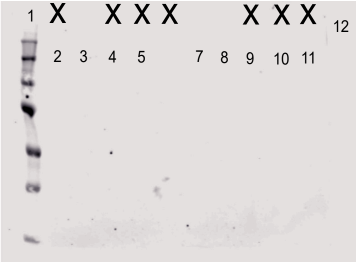

X = Not used in the study
1 = Molecular weight marker
2, 4, 5, 10, 11 = Empty well
3 = *P.g* bacteria
6 = *Pseudomonas aeuruginosa*
7 = *T.f* bacteria
8 and 9 = MAA-BSA
12 = *Aa*-HSP60
